# CarrierSeq: a sequence analysis workflow for low-input nanopore sequencing

**DOI:** 10.1186/s12859-018-2124-3

**Published:** 2018-03-27

**Authors:** Angel Mojarro, Julie Hachey, Gary Ruvkun, Maria T. Zuber, Christopher E. Carr

**Affiliations:** 1 Department of Earth, Atmospheric and Planetary Sciences, Massachusetts Institute of Technology, 77 Massachusetts Ave, E25-610, Cambridge, MA 02139 USA; 2 ReadCoor, Cambridge, MA USA; 3 Department of Molecular Biology, Massachusetts General Hospital, Boston, MA USA

**Keywords:** Nanopore sequencing, Low-input sequencing, Metagenomics

## Abstract

**Background:**

Long-read nanopore sequencing technology is of particular significance for taxonomic identification at or below the species level. For many environmental samples, the total extractable DNA is far below the current input requirements of nanopore sequencing, preventing “sample to sequence” metagenomics from low-biomass or recalcitrant samples.

**Results:**

Here we address this problem by employing carrier sequencing, a method to sequence low-input DNA by preparing the target DNA with a genomic carrier to achieve ideal library preparation and sequencing stoichiometry without amplification. We then use CarrierSeq, a sequence analysis workflow to identify the low-input target reads from the genomic carrier. We tested CarrierSeq experimentally by sequencing from a combination of 0.2 ng *Bacillus subtilis* ATCC 6633 DNA in a background of 1000 ng *Enterobacteria phage λ* DNA. After filtering of carrier, low quality, and low complexity reads, we detected target reads (*B. subtilis*), contamination reads, and “high quality noise reads” (HQNRs) not mapping to the carrier, target or known lab contaminants. These reads appear to be artifacts of the nanopore sequencing process as they are associated with specific channels (pores).

**Conclusion:**

By treating sequencing as a Poisson arrival process, we implement a statistical test to reject data from channels dominated by HQNRs while retaining low-input target reads.

## Background

Environmental metagenomic sequencing poses a number of challenges. First, complex soil matrices and tough-to-lyse organisms can frustrate the extraction of deoxyribonucleic acid (DNA) and ribonucleic acid (RNA) [1]. Second, low-biomass samples require further extraction and concentration steps which increase the likelihood of contamination [2]. Third, whole genome amplification may bias population results [3] while targeted amplification (e.g., 16S rRNA amplicon) may decrease taxonomic resolution [4]. To address these challenges, we have developed extraction protocols compatible with low-biomass recalcitrant samples and difficult to lyse organisms [5]. These protocols, developed using tough-to-lyse spores of *Bacillus subtilis*, allow us to achieve at least 5% extraction yield from a 50 mg sample containing 2 × 10^5^ cells/g of soil without centrifugation [6]. Furthermore, in order to avoid possible amplification biases and additional points of contamination, we have experimented with utilizing a genomic carrier (*Enterobacteria phage λ*) to shuttle low-input amounts of target DNA (*B. subtilis*) through library preparation and sequencing with ideal stoichiometry [7]. This approach has allowed us to detect down to 0.2 ng of *B. subtilis* DNA prepared with 1000 ng of Lambda DNA using the Oxford Nanopore Technologies (ONT) MinION sequencer. Here we present CarrierSeq, a sequence analysis workflow developed to identify target reads from a low-input sequencing run employing a genomic carrier.

## Implementation

CarrierSeq implements bwa-mem [8] to first map all reads to the genomic carrier then extracts unmapped reads by using samtools [9] and seqtk [10]. Thereafter, the user can define a quality score threshold and CarrierSeq proceeds to discard low-complexity reads [11] with fqtrim [12]. This set of unmapped and filtered reads are labeled “reads of interest” (ROI) and should theoretically comprise target reads and likely contamination. However, ROIs also include “high-quality noise reads” (HQNRs), defined as reads that satisfy quality score and complexity filters yet do not match to any database and disproportionately originate from specific channels. By treating reads as a Poisson arrival process, CarrierSeq models the expected ROIs channel distribution and rejects data from channels exceeding a reads/channels threshold (x_crit_).

### Quality score filter

**Fig. 1:**
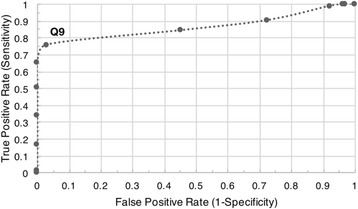
Receiver operating characteristic curve. Q9 provides a good threshold which discards the majority of low-quality and noise reads (0.76 True Positive Rate and 0.03 False Positive Rate) for carrier runs that are 99% Lambda DNA by mass. A perfect quality score threshold would plot in the top left of the ROC curve

The default per-read quality score threshold (Q9) was determined through receiver operating characteristic curve (ROC) analysis [13] of carrier sequencing runs of *B. subtilis* and Lambda DNA (Fig. 1). This threshold is best suited for Lambda carriers that are 99% library by mass and essentially function as a pseudo “lambda burn-in”. Therefore, the user is encouraged to define their own threshold based on their libraries’ quality control metrics (e.g., quality distribution, sequencing accuracy achieved, and basecaller confidence).

### Poisson sorting

Assuming that sequencing is a stochastic process, CarrierSeq is able to identify channels producing spurious reads by calculating the expected Poisson distribution of reads/channel. Given total ROIs and number of active sequencing channels, CarrierSeq will determine the arrival rate (λ = reads of interest/active channels). CarrierSeq then calculates an x_crit_ threshold (x_crit_ = poisson.ppf (1 – *p*-value), λ)) and sorts ROIs into target reads (reads/channel ≤ x_crit_) or HQNRs (reads/channel > x_crit_).

### Library preparation

Here we test CarrierSeq by analyzing carrier sequencing data from a library containing 0.2 ng of *B. subtilis* DNA prepared with 1000 ng of Lambda DNA using the Oxford Nanopore Technologies (ONT) ligation sequencing kit (LSK-SQK108). Following the standard Nanopore Lambda calibration or “burn in” protocol recommended for every new Nanopore user, *B. subtilis* DNA was used in place of the 3.6 kb positive control DNA. The library was then sequenced on a MinION Mark-1B sequencer and R9.4 flowcell for 48 h and basecalled using ONT’s Albacore (v1.10) offline basecaller.

## Results

### Sequencing

From the resulting 48 h of sequencing, we detected a total of 718,432 reads or 6.4 gigabases. Exactly 676,086 reads mapped to Lambda, 777 reads mapped to *B. subtilis,* and 41,569 reads mapped to neither.

### ROIs and sorting

**Fig. 2:**
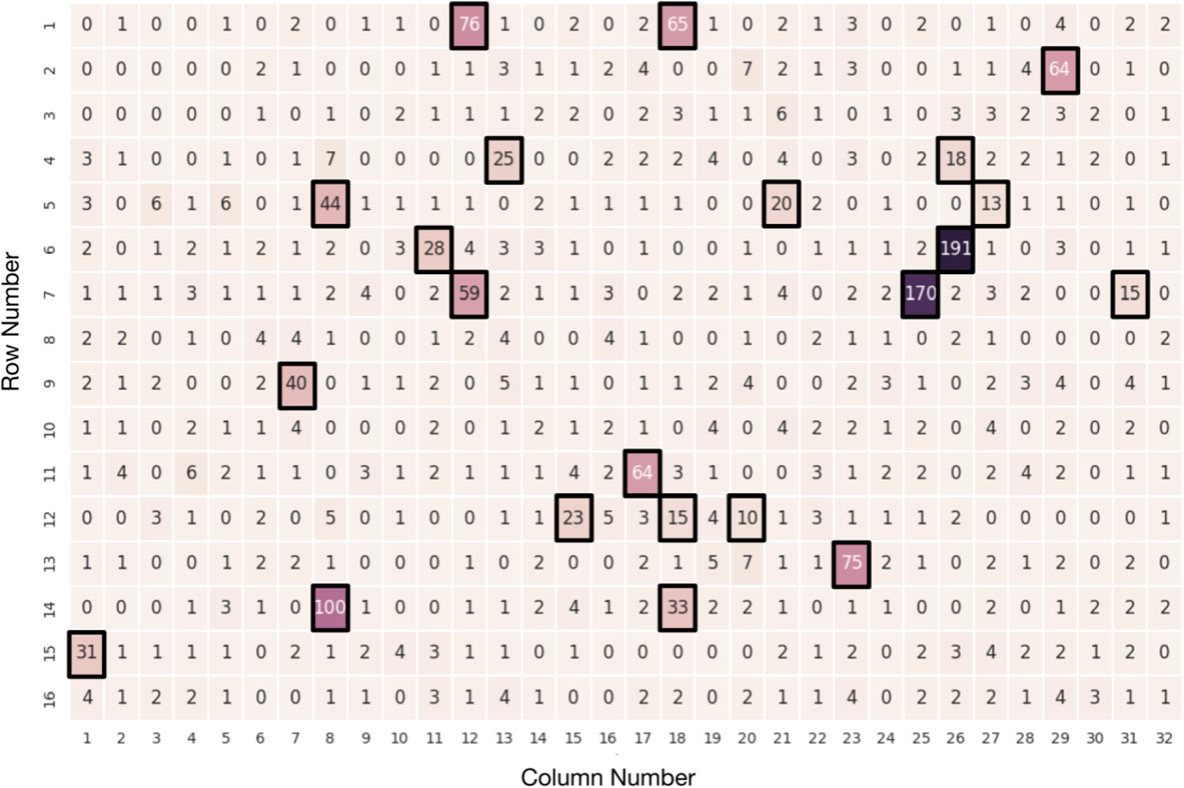
ROI Pore Occupancy. ROI read distribution across 512 sequencing channels. Assuming that sequencing is a stochastic process, we should expect a Poisson distribution of reads/channel. However, we discovered that overly productive channels not fitting the expected distribution model (e.g., up to 191 reads/channel, black boxes) produced spurious reads not belonging to the carrier, target, or known contamination. Here, channels producing more than 7 reads were identified as HQNR-associated

Applying the parameters *p* = 0.05 and q = 9, CarrierSeq identified 1811 ROIs and determined x_crit_ = 7. Therefore, channels producing greater than 7 reads were identified as HQNR-associated while channels producing less than or equal to 7 reads were identified as “good” channels (Fig. 2). CarrierSeq then sorted 1179 reads, including 1162 true negative reads (*real* HQNRs) and 17 false negative reads (*B. subtilis),* as likely HQNRs. The final 632 target reads consisted of 574 true positive reads (574 *B. subtilis* and 4 *Homo sapiens*) and 54 false positive reads (HQNRs). Overall, CarrierSeq identified 74% of all *B. subtilis* reads present. Moreover, from the discarded 203 *B. subtilis* reads, 186 were below Q9 while 17 originated from discarded HQNR-associated channels.

## Discussion

From experimenting with low-input carrier sequencing and CarrierSeq we observed that the abundance of HQNRs may vary per run, perhaps due to sub-optimal library preparation, delays in initializing sequencing, or other sequencing conditions. In addition, target DNA purity and lysis carryover (e.g., proteins) may conceivably contribute to HQNR abundance possibly due to pore blockages from unknown macromolecules that result in erroneous reads. While the cause or significance of HQNRs have yet to be determined, future work will focus on developing a method to identify HQNRs on a per-read basis. In contrast, the current approach discards entire HQNR-associated channels at the risk of discarding target reads. Moreover, some reads in non-HQNR-associated channels may also be artifacts. The ability to identify HQNRs on a per-read basis is especially important for metagenomic studies of novel microbial communities where HQNRs may complicate the identification of an unknown organism, or in a life detection application [6] where artefactual reads not mapping to known life could represent a false-positive.

## Conclusion

CarrierSeq was developed to analyze low-input carrier sequencing data and identify target reads. We have since deployed CarrierSeq to test the limits of detection of ONT’s MinION sequencer from 0.2 ng down to 2 pg of low-input carrier sequencing. CarrierSeq may be a particularly valuable tool for in-situ metagenomic studies where limited sample availability (e.g., low biomass environmental samples) and laboratory resources (i.e., field deployments) may benefit from sequencing with a genomic carrier.

### Availability and requirements

Project name: CarrierSeq.

Project home page: https://github.com/amojarro/carrierseq

Operating system(s): macOS and Linux.

Programming language: BASH and Python.

Other requirements: bwa, seqtk, samtools, fqtrim, Biopython, Docker (optional).

License: MIT.

Any restrictions to use by non-academics: None.

## Abbreviations

HQNR: High-quality noise reads
ONT: Oxford Nanopore Technologies
ROI: Reads of interest
